# Bone tissue and mineral metabolism in hereditary endocrine tumors: clinical manifestations and genetic bases

**DOI:** 10.1186/s13023-020-01380-1

**Published:** 2020-04-23

**Authors:** Davide Maraghelli, Francesca Giusti, Francesca Marini, Maria Luisa Brandi

**Affiliations:** Department of Experimental and Clinical Biomedical Sciences, University of Florence, Largo Palagi, 1, 50139 Florence, Firenze Italy

**Keywords:** Hereditary syndromic endocrine tumors, Bone clinical affections, Genetic bases

## Abstract

Inherited endocrine tumors are neoplasms of endocrine cells, transmitted via autosomal dominant germinal mutations. They present in two different forms: non-syndromic (patient has a single affected endocrine organ during his/her lifetime) or syndromic forms (multiple tumors in endocrine and non-endocrine organs during his/her lifetime).

In addition to their common tumoral manifestations, many of these diseases present clinical affection of bone tissues and/or mineral metabolism, both as secondary complications of primary tumors and as primary defects due to genetic mutation. To date, few studies have documented these bone complications, and there are no systematic reviews in this area.

We present a revision of medical literature about skeletal and mineral metabolism affections in inherited endocrine tumor syndromes, and studies, in cells and animal models, investigating the direct role of some genes, whose mutations are responsible for the development of endocrine tumors, in the regulation of bone and mineral metabolism.

## Background

In addition to their common manifestations, almost all inherited endocrine tumor syndromes present features of bone and mineral metabolism, either as secondary complications of primary tumors or as a primary consequence of gene mutation. There are few descriptions of these bone phenotypes in literature, and a review is still missing. This paper reviewed skeletal and mineral metabolism complications in hereditary endocrine tumor syndromes, focusing also on a description of the in vitro and in animal model studies investigating the direct role of genes, responsible for syndromes, in bone modeling and remodeling and/or in mineral metabolism homeostasis.

## Discussion

### Hereditary endocrine tumors

Endocrine tumors originate from specialized hormone-secreting cells. Most of these neoplasms specialize in synthesizing and secreting hormones (functioning tumors; FTs) and only a small portion lose the hormone-secretive ability (non-functioning tumors; NFTs). Few patients without a clinical family history develop endocrine tumors (sporadic forms), while most cases have mendelian heritability (inherited forms). Inherited forms can be non-syndromic (only a single affected endocrine organ during lifetime) or syndromic (multiple tumors in endocrine and non-endocrine organs during lifetime). All syndromes have an autosomal dominant inheritance.

Hereditary non-syndromic forms include familial primary hyperparathyroidism (FPHPT), familial medullary thyroid carcinoma (FMTC), familial isolated pituitary adenoma (FIPA), and familial acromegaly (FA).

Hereditary syndromic forms include Multiple Endocrine Neoplasia type 1, type 2 A and B and type 4 (MEN1, MEN2A, MEN2B and MEN4), von Hippel Lindau (VHL), hereditary Paraganglioma/Pheochromocytoma syndromes (PGL/PCC), Hyperparathyroidism-Jaw Tumor syndrome (HPT-JT), Cowden syndrome (CS), Carney Complex (CNC), Tuberous Sclerosis (TSC), and Neurofibromatosis type 1 (NF1).

### Multiple Endocrine Neoplasia type 1

MEN1 is characterized by: 1) adenomas of parathyroid/adenomas of parathyroid (affecting over 90% of patients by the age of 50 and causing primary hyperparathyroidism (PHPT), which can be associated with hypercalcemia); 2) adenomas of adenohypophysis (mainly prolactin-secreting adenoma or, more rarely, non-functioning macroadenomas); 3) neuroendocrine tumors of gastro-entero-pancreatic tract (GEP-NETs) [1]; (Table 1).

**Table 1:** Bone phenotypes in hereditary endocrine tumors, and possible molecular involvement of responsible genes in the regulation of bone and mineral metabolism

Syndrome	Main clinical manifestations of the syndrome	Skeletal manifestations	Responsible gene(s)	Role of genes in bone and/or mineral metabolism
MEN1	PHPT, hypophyseal adenomas, GEP-NETs, carcinoids, adrenal-cortical tumors	Osteoporosis and/or osteopenia	*MEN1* (11q13)	Regulation of osteogenesis, promotion of osteoblastic differentiation
MEN2	MTC, PHEO, PHPT, CLA, HD	Marfanoid habitus, pectus excavatum, pes cavus, equino-varus foot, femoral epiphysiolysis, kyphosis, scoliosis and increased joint laxity (MEN2B)	*RET* (10q11.21)	Possible up-regulation of chondromodulin-1, which promotes cartilage deposition and inhibits bone deposition
MEN4	PHPT, hypophyseal adenomas, adrenal, renal and reproductive organs tumors	Osteoporosis and/or osteopenia	*CDKN1B* (12p13.1-P12)	Regulation of longitudinal bone growth and endochondral ossification
VHL	Retinal, cerebellar and medullary hemangioblastomas, RCC, PHEO	No manifestation reported to date	*VHL* (3p25.3)	Vascularization in endochondral and membranous ossification
PGL/PCC syndromes	Secreting PGL e PHEO, HNPGL	No manifestation reported to date	*SDHx* (1q21; 1p36.1-p35; 11q23; 11q31.1)	No role of the SDHx genes on bone metabolism reported to date
HPT-JT	PHPT, ossifying fibromas of the maxilla and mandible, renal tumors and adenomatous polyps of the uterus	Osteoporosis and / or osteopenia, ossifying fibromas of the maxilla and mandible, osteitis fibrosa cystica	*HRPT2 / CDC73* (1q31.2)	Transcriptional repression of osteoprogenitor cells necessary for cellular survival and regulation of cell differentiation and bone homeostasis
CS	Multiple hamartomas, susceptibility to malignant tumors, skin and facial changes, CNS abnormalities and fibrocystic breast disease, thyroid carcinoma	Macrocephaly, bone cysts, thoracic kyphosis, kyphoscoliosis, pectus excavatum, large hands and feet, syndactyly, maxillary and scapular hypoplasia	*PTEN* (10q23.3)	Regulation of osteoblastic apoptosis/survival, osteoblastic differentiation regulation, indirect regulation of chondrocyte adaptation to hypoxic stress
CNC	Heart, endocrine, cutaneous and neural myxomatous tumors, pigmented lesions of skin and mucous membranes	Osteochondromyxomas	*PRKAR1A* (17q22–24); or possible mutation in 2p16	Osteoblastic differentiation and promotion of osteogenesis
TSC	CNS, cardiac, renal, cutaneous, ocular and pulmonary hamartomas; pancreatic NETs, pituitary and parotid adenomas	Metacarpal and metatarsal bone cysts, sclerotic bone lesions	*TSC1* (9q34) and *TSC2* (16p13)	No studies are available to document a direct role of TSC1 and TSC2 in bone metabolism
NF1	Café-au-lait spots, Lisch nodules, neurofibromas, neurofibrosarcomas, gliomas, PHEO, myeloid leukemia and GEP-NETs	Kyphoscoliosis, macrocephaly, sphenoid wing dysplasia, congenital curvature, and tibial pseudoarthrosis	*NF1* (17q11.2)	Regulation of osteogenic proliferation and differentiation, reduction of expression of osteopontin (calcification inhibitor) in pre-osteoblastic MSC

**Footnotes: PHPT**: Primary HyperParaThyroidism; **GEP-NETs**: GastroEnteroPancreatic NeuroEndocrine Tumors; **MTC**: Medullary Thyroid Carcinoma; **PHEO**: PHEOchromocytoma; **CLA**: Cutaneous Lichen Amyloidosis; **HD**: Hirschsprung Disease; **RCC**: Renal Cell Carcinoma; **PGL**: ParaGangLioma: **HNPGL**: Head and Neck ParaGangLioma.

MEN1 is caused by germinal heterozygote inactivating mutations of the *MEN1* tumor suppressor gene, encoding a nuclear protein (menin) that exerts key functions in the regulation of important biological processes (cell cycle, DNA repair, apoptosis, gene transcription, and osteoblast differentiation) [2]; (Table 1).

Menin interacts with many different proteins and is involved in the regulation of numerous molecular pathways, opening the possibility to have a wide spectrum of potential targets for molecular therapy of the syndrome. To date, in vivo studies on pancreatic NETs have shown efficacy towards epigenetic modulators such as bromo- and extra terminal domain (BET) inhibitors, Wnt pathway targeting β-catenin antagonists, VEGF-signalling and mammalian target of rapamycin (mTOR) antagonists [3].

#### Bone phenotype in MEN1

Osteoporosis and osteopenia are frequent, early complications in MEN1 patients (Table 1]; reduction of bone mass is a common early clinical sign in MEN1 women by the age of 35 [4]. PHPT is the main cause of bone loss. The anticipated development of PHPT (20–30 years) can significantly interfere with the achievement of normal peak of bone mass, and untreated prolonged excessive secretion of parathyroid hormone (PTH) increases bone resorption, leading to early loss of cortical and trabecular bone. Early treatment of symptomatic PHPT, by successful subtotal parathyroid surgery, reduces skeletal damage, with significant improvement to BMD in the first 12 months [4].

Additional conditions may be considered risk factors for osteoporosis, such as hypogonadism, growth hormone deficiency, hypocortisolism due to pituitary disorders, as well as malabsorption caused by surgical resection of the proximal tract of the small intestine [5].

Despite a milder biochemical presentation, bone alterations in MEN1 PHPT are more severe than sporadic PHPT [6], indicating that bone phenotype in MEN1 is not just a secondary consequence of PHPT and suggesting a direct role of the *MEN1* gene in bone physiology regulation.

Surgical removal of parathyroid adenomas, responsible for PHPT is the treatment of choice for restoring normal PTH secretion and serum level of calcium; parathyroidectomy demonstrated to normalize bone and mineral metabolism parameters. Medical therapy with cinacalcet demonstrated to be effective to control PTH over-secretion, and subsequent hypercalcemia, in patients who manifest PHPT recurrence or persistence despite one or more reoperations and in patients who are not suitable for parathyroidectomy. This calcimimetic molecule increases the sensitivity of the calcium-sensing receptor to extracellular calcium and induces a decrease in PTH secretion being able to normalize parathyroid functionality and, thus, bone turn-over.

#### Role of the *MEN1* gene in bone phenotypes

Several studies, performed in MEN1 mice models and in cells, have confirmed direct important functions of wild type menin in regulation of osteogenesis and maintenance of bone mass. Heterozygous knock-out (KO) mice for the *Men1* gene (*Men1*^*+/−*^) showed a significant reduction in BMD, trabecular bone volume and cortical bone thickness, compared to control mice [7]. In *Men1*^*+/−*^ mice, the number of osteoblasts and osteoclasts and rate of mineralization were reduced, while the number of osteocytes was increased [7]. Conversely, transgenic mice induced to over-express menin showed an increase in bone mass, number of osteoblasts, and rate of mineralization [7].

Menin directly interacts with JunD, a transcription factor involved in negative control of cell proliferation and regulation of bone metabolism, repressing its activity [8]. JunD-deficient mice showed increased bone mass and trabecular number, associated with increased osteoblast activity [9]. The upregulation of JunD, caused by menin deficiency, could directly alter bone metabolism in MEN1 patients.

Menin has been shown to balance the commitment of mesenchymal stem cells (MSCs) to osteogenic or myogenic lineages. In response to BMP2, menin enhances the transcriptional activity of SMAD1/5factors, promoting osteoblastic differentiation of MSCs. Moreover, under the effect of TGF-β1, menin reinforces repression of myogenic differentiation mediated by SMAD3/TGFβ1 [10]. When osteoblast activity is required, the expression of menin, in MSCs, is over-regulated. A reduction of wild type menin expression in these precursors can induce myogenesis [10].

Menin acts as direct positive regulator of miR-26a [11], a microRNA that negatively regulates the expression of SMAD1 protein during osteoblastic differentiation of MSCs [11].

Wild type menin directly increases the transcriptional activity of two steroid receptors exerting an important role in bone modeling and remodeling [the vitamin D receptor (VDR) and estrogen receptor α (ERα)]. In parathyroid cells, vitamin D inhibits PTH transcription and cell proliferation; a reduction of VDR activity, following the loss of menin, could represent a facilitation for the growth of parathyroid cells and adenoma development [12].

### Multiple Endocrine Neoplasia type 2

MEN 2 includes two clinical subtypes: 2A (MEN2A) and 2B (MEN2B) [13]. MEN2 tumors are medullary thyroid carcinoma (MTC), pheochromocytoma (PHEO) and, only in MEN2A, parathyroid adenoma/hyperplasia, associated with PHPT [14]; (Table 1].

MEN2 is caused by germinal heterozygote activating point mutations of the proto-oncogene *RET* (REarranged during Transfection; 10q11.21) (Table 1], encoding the homonym trans-membrane tyrosine kinase receptor (RET) responsible for positive regulation of cell growth. Different mutations are responsible for different clinical phenotypes. MEN2A patients have mutations at codons 634 (85% of cases), 609, 611, 618 and 620 [15]. Over 90% of MEN2B patients present the de novo M918T mutation [15–17]; mutation at codon 883 (A883F) affects about 5% of MEN2B patients.

Since the early 2000s, targeted medical therapies have been introduced in MEN2 patients, especially designed for the treatment of MTC. They consists of anti-angiogenic drugs and multi-targeted tyrosine-kinase inhibitors, not specifically directed against RET; for this reason the overall effectiveness is reduced and the incidence of drug resistance and side effects are increased [18]. Recently, quinazoline compounds, specific inhibitors of the RET thyrosine kinase pathway showed mild to significant anticancer efficacy in some tumor cell lines, including thyroid cancer, suggesting a possible future therapeutic potential in MEN2 [19].

#### Bone phenotype in MEN2

Skeletal affection in MEN2A is caused by PHPT, consisting in early reduction of BMD and increased risk of fracture [20, 21]. PTH normalization, following surgical removal of parathyroid adenoma, reverts the bone mass loss [19]. PHPT in MEN2A is usually milder than in MEN1 and BMD loss is less common and less severe [22].

MEN2B phenotype is characterized by typical musculoskeletal abnormalities, including marfanoid habitus with tall and slender body, long limbs, ogival palate, and long and thin face with prognathism. Bone abnormalities, such as pectus excavatum, hollow foot, equinovarus foot, femoral epiphysiolysis, kyphosis, scoliosis and increased joint laxity, are also present [23]; (Table 1).

For MEN2A-PHPT therapy with cinacalcet can be considered, with the same indication of MEN1. Not specific RET-targeted drugs for correction of bone affections in MEN2B patients are available or under evaluation, yet.

#### Role of the *RET* gene in bone modelling

Skeletal anomalies, manifesting in MEN2B form, can be ascribed to the specific *RET* mutations, affecting the intracellular domain of the protein and responsible for MEN2B phenotype. In fact, up-regulation of chondromodulin-1 (CHM1), a cartilaginous and bone growth regulator protein, normally expressed in proliferating and pre-hypertrophic chondrocytes of growth plates, was observed only in MTC samples of MEN2B patients. Increased transcription of *CHM1* could be a direct result of signaling alterations secondary to the RET intracellular mutation, suggesting a possible relationship, not yet determined, between the two genes [24]. All MEN2B patients with skeletal anomalies had high expression of CHM1 [24]; (Table 1). In bone explants, the over-expression of CHM1 promotes cartilage deposition and inhibits bone formation. *Chm1* mutant mice showed increased bone deposition [24]. Altered expression of CHM1 is suspected to alter the bone growth plate, leading to the skeletal abnormalities observed in MEN2B patients.

Activating mutations of the intracellular domain of RET could also be suspected to be responsible for the overexpression, in MEN2, of stanniocalcin-1 (STC1), a protein involved in the regulation of bone metabolism, by acting as an autocrine/paracrine regulator of calcium and phosphate homeostasis, with a role in early skeletal patterning and joint formation, [25]. The high expression of STC1 during embryogenesis could be associated with the marfanoid skeletal phenotype.

### Multiple Endocrine Neoplasia type 4

MEN4 is characterized by multiple parathyroid tumors with associated PHPT (81% of cases) and adenohypophysial tumors (42%; ACTHoma with Cushing syndrome, GHoma, PRLoma, and non-functioning pituitary adenoma) (Table 1). Adrenal, renal and reproductive organ tumors, gastric and bronchial carcinoids and Zollinger-Ellison syndrome are also documented [26].

MEN4 is caused by inactivating heterozygous mutations of the *CDKN1B* tumor suppressor gene (12p13.1) (Table 1], encoding the p27^kip1^ protein, a CDK2 cyclin-dependent kinase inhibitor, which acts as a negative regulator of cell cycle progression at G1-S checkpoint [27]. Mutations create an altered or truncated p27^kip1^ that is localized within the cytosol instead of the nucleus (contrary to the wild type counterpart), where it is not able to bind CDK2 [27].

Currently, only 19 cases of MEN4 have been described worldwide and, thus, no sufficient data are available about the efficacy of medical therapies for this syndrome. Therapeutic approaches follow the same guidelines of MEN1, and no specific research about possible gene therapies in patients with *CDKN1B* mutations has been carried out yet. Application of p27 gene therapy was tested in human glioblastoma cells through CDKN1B-containing adenoviral vector transfection, showing promising anti-proliferation effects [28]. This finding can be a starting point for future research in p27 gene therapy also for MEN4 patients.

#### Bone phenotype in MEN4

Given the small number of MEN4 cases reported to date, no targeted studies on bone complications have been specifically conducted and data are not yet available in literature. However, since MEN4 presents a clinical phenotype overlapping that of MEN1, it is highly probable that PHPT-related hypercalcemia can alter bone metabolism and reduce BMD value the same way we find in MEN1 patients (Table 1).

#### Role of the *CDKN1B* gene in bone and mineral metabolism

Studies on *Cdkn1b*-KO mice showed increased growth in animals lacking p27^Kip1^-mediated CDK2 inhibition, with a progressively growing effect with one or two silenced alleles. At cellular level, *p27*^*−/−*^ mice showed increased proliferative activity of bone marrow cells and formation of numerous osteoblastic colonies. In the growth plate of developing organism, the expression of p27^Kip1^ is about two times lower than the surrounding bone cells of the epiphyseal and metaphyseal bone; this guarantees longitudinal bone growth (Table 1). p27^Kip1^ expression inhibits chondrocyte proliferation within growth plates and induces osteoblastic-like cell differentiation, via a BMP4-induced signaling pathway. Functional studies have shown that p27^Kip1^ modulates the regulation of skeletal development and osteoblastic bone formation with a mechanism that implies its direct interaction with the PTH-related peptide (PTHrP) [29]. Homozygous mutant murine models for both p27^Kip1^ and Pthrp (*p27*^*−/−*^*/Pthrp KI*) showed an increase in body weight, lifespan and growth of long bones, due to a higher proliferative state of growth plate, when compared to wild type and mutant models for Pthrp (*Pthrp KI*) or p27^Kip1^ (*p27*^*−/−*^) [29]. BMD, cortical, epiphyseal and trabecular bone volume, number of osteoblasts and bone areas positive for type I collagen and osteocalcin, were all significantly increased in *p27*^*−/−*^ mice and significantly reduced in *Pthrp KI* and *p27*^*−/−*^*/Pthrp KI* mice, compared to wild-type mice. The same parameters significantly increased in *p27*^*−/−*^*/Pthrp KI* mice compared to *Pthrp KI* mice. Pthrp, Igf-1 and Bmi-1 expression was increased in *p27*^*−/−*^ mice and reduced in *Pthrp KI* and *p27−/−/Pthrp KI* mice with respect to wild type; expression of these three genes was increased in *p27*^*−/−*^*/Pthrp KI* mice compared to *Pthrp KI* mice [29]. The number of colonies-forming units-fibroblasts (CFU-f) and CFU-f positive for alkaline phosphatase (ALP) increased in *p27*^*−/−*^ mice with respect to wild-type mice, and in *p27*^*−/−*^*/Pthrp KI* mice compared to *Pthrp KI* mice. Conversely, there was a reduction of CFU-f in *Pthrp KI* and *p27*^*−/−*^*/Pthrp KI* mice compared to wild-type. These data indicate that p27^kip1^ may have an effect on recruitment and differentiation of bone marrow MSCs (BM-MSCs) [29]. *p27*^*−/−*^ mice also showed an increase of RANKL/OPG ratio, presumably responsible for over-activation of osteoblast activity [29]. p27^kip1^ may be directly involved in the regulation of skeletal growth through its direct interaction with the C-terminal region and nuclear localization signals (NLSs) of PTHrP. This interaction leads to the inhibition of p27^kip1^, enhancing the proliferation of chondrocytes and BM-MSCs and their differentiation into mature and active osteoblasts. The deletion of p27^kip1^ in *Pthrp KI* mice appears to partially compensate for bone growth defect and restore osteoblastic differentiation, increasing endochondral ossification and osteogenesis (Table 1), suggesting a possible future use of NLS and C-terminal regions of PTHrP to promote bone growth [29].

### Von Hippel-Lindau syndrome

VHL predisposes development of various malignant and benign tumors, specifically, retinal, cerebellar and spinal hemangioblastomas, renal carcinomas (RCC), PHEO, and pancreatic tumors (Table 1). Hemangiomas of the adrenal glands, lungs and liver, and multiple cysts of the pancreas and kidneys, have been also described [30].

VHL is caused by inactivating mutations in the tumor suppressor gene *VHL* (3p25.3) (Table 1), encoding the homologous protein (pVHL), which can be totally absent or inactive [31].

Normally, pVHL negatively regulates the hypoxia inducible factor 1α (HIF-1α) responsible for cellular responses to hypoxia, including development of neoangiogenesis and induction of cell proliferation. Loss of wild type pVHL prevents degradation of HIF factors and induces responses to hypoxia, even when not physiologically required, inducing cell growth and formation of new vessels, thus favoring tumorigenesis [31].

Molecular therapy in VHL is precisely targeted towards genes regulated by HIF-1α, such as VEGF, PDGF-β and TGF-α, which have a fundamental role in tumorigenesis and angiogenesis. However, if anti-VEGF drugs, as bevacizumab, or multi-targeted receptor tyrosine kinase inhibitors, as sunitinib, have shown proven efficacy towards sporadic forms of cerebellar hemangioma, pancreatic NETs and RCC, a specific role of these agents in VHL patients, although used, is less clear [32]. Promising drugs in the future could be mTOR inhibitors: mTOR kinase in fact plays a critical role in the transcription and translation of HIF-2α; these inhibitors have shown positive activity in patients with sporadic pacreatic NETs but a possible benefit in patients with VHL mutations has not yet been sought [32].

#### Bone phenotype in VHL and role of the *VHL* gene in bone tissue

No clinical manifestations affecting bone and/or mineral metabolism were described in VHL patients (Table 1). No direct effects of VHL-related tumors on bone and mineral metabolism have been demonstrated. Indeed, only the complete loss of pVHL in cartilage cells can alter the correct bone growth and metabolism, as reported by studies in murine models with selective inactivation of the *Vhlh* gene only in the cartilaginous cells (Vhlh-null mice) [33]. While in VHL the somatic loss of heterozygosity of *VHL* gene has been described only in retinal and CNS hemangioblastomas, RCC, PHEO, and pancreatic tumors. This fact can explain why VHL patients do not manifest bone alterations, despite the direct role of pVHL in bone modelling and endochondral ossification.

### Hereditary Paraganglioma-Pheochromocytoma syndromes

PGL-PCCs are characterized by the presence of paragangliomas (PGLs) from the skull to the pelvis, and PHEOs (Table 1). Sympathetic PGLs and PHEOs secrete catecholamines, which are directly responsible for clinical symptoms, while parasympathetic PGLs are most often non-secretory, located in the head and neck, and symptoms depend on the compression of surrounding vascular-nervous structures [34].

PGL-PCCs are caused by germline mutations of genes encoding components of the enzymatic succinate dehydrogenase complex, or complex 2, of the mitochondrial respiratory chain [35]; (Table 1). Different genes are responsible for specific PGL-PCC subtypes: *SDHD* (11q23) in PGL-PCC1, *SDHC* (1q21) in PGL-PCC3, *SDHB* (1p36.1-p35) in PGL-PCC4, and *SDHAF2* (*SDH5*) (11q31.1) in PGL-PCC2. Homozygous mutations of *SDHA* gene (5p15) cause a clinical phenotype (Leigh syndrome) characterized by degenerative myeloencephalopathy.

The possible targets of molecular therapy in the PGL-PCCs syndrome belong to the hypoxia-associated signal pathway. Indeed, mutations of *SDHx* genes induce energy metabolism disorders and succinate accumulation, the last inhibits the activity of PHD2 and leads to increased activation of HIF-α. SDHx-associated tumors are usually highly vascularized, thus, anti-angiogenic drugs can represent an effective treatment. A clinical study and case report demonstrated that oral sunitinib was effective in treating PCC/PGL SDHx-related [36]. Ethacrynic acid and idarubicin are other HIF inhibitors that may be studied in the future as specific target drugs in this hereditary syndrome [36].

#### Bone affections in PGL-PCC

No bone and/or mineral manifestations have been described in patients with PGL-PCC syndromes (Table 1). The only “intersection” between these syndromes and the skeleton are bone metastases in rare malignant forms of the disease, mainly localized in the rachis [37]. To date, no studies on animal models with mutations of *SDHx* genes and bone diseases, nor specific studies that correlate the gene encoding components of the enzymatic succinate dehydrogenase complex or complex 2 of the mitochondrial respiratory chain with bone and/or mineral metabolism, have been published (Table 1).

### Hyperparathyroidism-jaw tumor syndrome

HPT-JT syndrome is characterized by synchronous or metachronous onset of PHPT, ossifying jaw fibromas, renal tumors, adenomatous uterine polyps (Table 1), and an increased risk of renal hamartomas, adult Wilms tumors and parathyroid carcinoma [38]. PHPT occurs in about 95–100% of patients, due to single or multiple parathyroid adenoma, often recurrent after partial surgery [39]. PHPT can cause kidney stones and urinary tract infections [40].

HPT-JT is caused by inactivating germinal mutations of the *HRPT2/CDC73* tumor suppressor gene (1q31.2) [41]; (Table 1), encoding a nuclear protein called parafibromin, a component of the PAF1 complex, that is responsible for H3K4 histone trimethylation and H3K79 histone methylation. The interaction of parafibromin with this complex depends on its C-terminal domain, which is suppressed in about 80% of clinically relevant mutations. Mutations of *CDC73* also cause *CDC73*-related isolated familial hyperparathyroidism and *CDC73*-related parathyroid carcinoma [40].

To date, there are no molecular targeted drugs acting on the CDC73/parafibromin pathway and effective for the treatment of HPT-JT.

#### Bone phenotype in HPT-JT

About 40% of HPT-JT patients develop ossifying jaw fibromas [40]; (Table 1), microscopically consisting of bone trabeculae with inhomogeneous fibrocellular stroma and mineralized matrix. These tumors may occur several decades before the development of hypercalcemia, suggesting that their development is not directly related to the increase of serum calcium [42]. Risk of post-surgical recurrence has been reported [43].

HPT-JT patients manifest PHPT with severe hypercalcemia if not diagnosed and treated early, resulting in possible development of severe bone resorption, osteopenia/osteoporosis, bone pain, elevated risk of fragility fractures, hypercalciuria, and nephrolithiasis (Table 1). Severe and prolonged PHPT (mainly from parathyroid carcinoma) alters bone metabolism and may give rise to multiple brown tumors (osteitis fibrosa cystica) (Table 1). They normally regress after normalization of serum calcium and PTH, following surgical removal of parathyroid tumor(s) [44].

#### Role of the *CDC73* gene in bone phenotype

Direct functions of parafibromin in bone tissue were studied by using murine models with a selective conditional deletion of the *Cdc73* gene in MSCs, mature osteoblasts and osteocytes. Homozygous deletion of *Cdc73* in MSCs resulted in embryos non-presenting mesenchymal development of the internal organs. The immunohistochemical staining of inactive and active caspase-3 revealed a high rate of apoptosis of MSCs [45]. Conversely, selective homozygous elimination of *Cdc73* in mature osteoblasts and osteocytes generated mice with normal life span, but increased cortical and trabecular bone [45]. This increment is associated with the presence of large cortical pores actively undergoing bone remodeling. The femoral cortical bone contained osteocytes with large amounts of cytosol and a high rate of apoptosis. Genes up-regulated in bone tissue in the absence of *Cdc73* are components of the extracellular bone matrix (type 1 collagen, integrins, aggrecan, matrilin, ligand of chemokines 2 and 10), while down-regulated genes are related to transcription and mitotic processes (*Sept10, Anapc13, Med16, MAPK*, *Rab1b*, and *Elk1*) [45]. A hypothesis is that, in MSCs, parafibromin acts as a transcriptional repressor, necessary for cell survival and involved in regulation of cell differentiation and bone homeostasis [45]; (Table 1).

### Cowden syndrome

CS is characterized by multiple hamartomas and increased risk of developing malignancies, skin and facial changes (trichilemmoms, papillomatous papules, and acral keratoses), CNS abnormalities, and fibrocystic breast disease [46]; (Table 1). The most frequent malignant tumors are ductal adenocarcinoma of the breast and papillary and follicular carcinoma of the thyroid. Polyposis of the gastro-intestinal tract is frequent (70–85%) [46].

*PTEN* (Phosphatase and TENsin homolog; 10q23.31), a tumor suppressor gene encoding a lipid phosphatase protein that negatively regulates the PI3K/AKT/mTOR signaling pathway, is the responsible gene [47–49]; (Table 1). Loss or reduction of PTEN activity results in increased phosphorylation of key cellular proteins, which can lead to uncontrolled cell cycle progression [50]. Over 80% of CS patients have a PTEN mutation [51]. Mutations in *SDHB, SDHD* or *KLLN* genes were recently identified in CS patients, with or without *PTEN* mutations [52].

No target therapies have been approved for CS patients but the upregulation of the PIK3/AKT/mTOR pathway suggests a role for therapies targeting this pathway [32]. Recently, a pilot study of sirolimus, a mTOR inhibitor, in 18 CS patients has shown that this drug is well tolerated in these subjects, and its administration is associated with improvement of symptoms, skin lesions, cerebellar function, and decreased mTOR signaling [53]. Selective PI3K beta inhibitors may have better activity and tolerability in patients with CS and other trials are investigating their role in patients with PTEN deficient advanced tumors [32].

#### Bone phenotype in CS

Several skeletal abnormalities has been reported in CS patients, including increased cranial size, bone cysts, thoracic kyphosis, kyphoscoliosis, pectus excavatum, large hands and feet, syndactyly, mandibular and maxillary hypoplasia, hypoplasia of shoulder blades, and osteosarcoma [46, 54, 55]; (Table 1). Data on the frequency of these abnormalities in CS patients are still lacking and a link with mutations of the *PTEN* gene has not been found [46].

#### Role of the *PTEN* gene in bone phenotype

Mice with osteoblastic precursors selectively lacking the *Pten* gene showed progressive and dramatic BMD increase over lifetime [56]; (Table 1). Pten-lacking osteoblastic precursors had greater differentiation potential than wild type counterparts, due to higher expression of beta catenin and subsequent activation of the Wnt signaling pathway. Pten-null osteoblasts expressed higher levels of osteogenic markers (ALP, osteocalcin, osteoprotegerin), associated with increased mineralization [57]; (Table 1).

A reduction of apoptosis has been reported in Pten^−/−^ chondrocytes of the growth plate, due to loss of Pten-derived hyper-activation of Akt [58]. Pten positively regulates the PI3K/Akt signaling pathway, which controls HIF-1α–mediated adaptation to hypoxic stress. Failure to express Pten induces stress of the endoplasmic reticulum and causes up-regulation of hypoxia-induced genes, including HIF-1α, [58]; (Tab.1). PTEN function in chondrocytes is essential for inhibiting dyschondroplasia; this could explain how the loss of PTEN protein has a direct pathogenic role in skeletal manifestations observed in CS patients [58].

### Carney complex

CNC is characterized by heart mixomas, neural tumors and neoplasms of the endocrine system, and a variety of pigmented lesions of the skin and mucous membranes in typical sites (lips, conjunctiva, genital mucosa) [59]; (Tab.1). CNC has a remarkable clinical heterogeneity with generational leaps and prevalent maternal inheritance.

Linkage analysis studies on affected families have identified two genetic loci associated with CNC at 2p16 and 17q22–24 [60]; (Table 1). Based on these genetic alterations, CNC was subdivided into two subtypes: CNC1 (mutation on *PRKAR1A* gene at 17q22–24) and CNC2 (mutation at 2p16 locus). Approximately 60% of CNC patients bear an inactivating germline mutation of the *PRKAR1A* tumor suppressor gene, encoding the I-alpha regulatory subunit of cAMP-dependent protein kinase (PKA) [61].

The cAMP analogue 8-Cl-adenosine has been shown in vitro to inhibit proliferation and promote apoptosis of cancer cells [62] and it could serve as a therapeutic agent in patients with CNC-related tumors.

#### Bone phenotype in CNC

A significant percentage of CNC patients develop bone tumors (osteochondromyxomas) (Table 1), characterized by layers of cells growing in macro- or micro-lobular patterns in a loose mixoid matrix, consisting of mesenchymal, cartilaginous, bone, and fibrous elements [63]. These tumors, prevalently located in the nasal region and diaphysis of tibia and radius, are usually painless and, therefore, detected during imaging evaluation for mass side effects [64, 65].

#### Role of the *PRKAR1A* gene in bone phenotype

*Prkar1a*^*+/−*^ mice showed large, hypocellular, bone lesions, composed of polygonal cells, with bone trabeculae surrounded by normal osteoblasts [63]. These mice developed bone tumors like human osteochondromyxomas, originating by MSCs differentiating into the osteoblastic line, although they appear arrested in a state of incomplete differentiation, as shown by the persistence of chondrocyte markers. Tumor cells show higher PKA activity and hyper-responsiveness to growth promoting effects of PKA activating agents (cAMP). Therefore, both increase in bone turnover and observed tumorigenesis are probably attributable to hyper-responsiveness to cAMP [66]. Analysis of gene expression of tumoral osteoblasts revealed reduced expression of osteoblastic differentiation markers and increased expression of local growth factors, including members of the Wnt signaling pathway [63].

The PKA signaling pathway is essential for bone mineralization in vitro and in vivo [66]. Loss of function and expression of the *PRKAR1A* gene leads to PKA level increase. Over-activation of PKA signaling causes fibrous dysplasia, an immature expansion of osteoblast precursors leading to structurally immature and hyper-proliferative bone. Expression of the I-alpha regulatory subunit of PKA increases during osteoblastic differentiation, suggesting its direct positive effect on the promotion of osteogenesis [66]. Conversely, the suppression of *PRKAR1A* gene expression could make cells refractory to the signals of osteogenic differentiation [66; Table 1). KO mice for *Prkar1a* showed significant suppression of bone formation and reduced expression of osteoblastic markers, such as osteocalcin and osteopontin. The loss of *Prkar1a*-encoded protein increases the activity of PKA, which inhibits the transcription of Runx2 and its action on the target genes, and negatively regulates osteoblast differentiation [66]. The PKA signaling pathway, a key signal transduction pathway downstream of the PTH receptor (PTH1R) and PKA hyper-activation, resulting from the loss of *PRKAR1A*, could influence the PTH response [66].

PRKAR1A haploinsufficiency induces cyclooxygenase-2 (COX2) activation and prostaglandin E2 (PGE2) overproduction, associated with abnormal proliferation of bone stromal cells of CNC osteochondromyxomas. Administration of celecoxib, a COX2 inhibitor, in mice with PKA defects decreased PGE2 level and reduced the proliferation of bone stromal cells, resulting in important reduction of osteochondromyxoma growth and better organization of the cortical bone adjacent to the mass [67]. This pivotal study suggests the possibility to use this drug for the treatment of bone affections in CNC.

### Tuberous sclerosis complex

TSC is a complex genetic disorder characterized by the growth of numerous noncancerous (benign) nodular tumors (hamartoma) in the CNS (giant cell astrocytoma, cortical tubers, subependymal nodules), heart (rhabdomyomas), kidneys (angiomyolipomas and cysts), skin (hypo-pigmented spots), lungs (lymphangioleiomyomatosis), and retina. Associations with pancreatic NETs (especially insulinomas), pituitary and parotid adenomas have also been described [68–70].; (Table 1).

TSC is caused by loss-of-function mutations, either of the *TSC1* gene (9q34), encoding hamartin, or the *TSC2* gene (16p13), encoding tuberin [71]; (Table 1), with over half of the cases arising from non-inherited embryonal mutations. Both genes are tumor suppressors. Loss of one of the encoded proteins is sufficient for development of TSC, leading to a complete loss of hamartin-tuberin complex activity, with no inhibitory modulation on the mTOR signaling cascade and subsequent alteration of nutritional signal transduction and stimulation of cell growth [68, 72].

mTOR inhibitors (sirolimus, everolimus) are used in the clinical practice of TSC patients for the treatment of enlarging subependymal giant cell astrocytomas, renal angiomyolipomas > 4 cm, or > 3 cm and growing rapidly, symptomatic cardiac rhabdomyomas and facial angiofibromas [73]. Recently, a study in a murine model of TSC with embryonic loss of Tsc1 in brain neurons, demonstrated the therapeutic efficacy of hamartin transfection by adenovirus vector, resulting in the normalization of neuron size and a decrease in markers of mTOR activation, with no side effects. Future studies are needed to evaluate the efficacy of this genetic therapy in humans [74].

#### Bone phenotype in TSC

Skeletal manifestations have been reported in TSC, mainly bone cysts in metacarpal and metatarsal bones, sclerotic bone lesions (SBL), and fibrous dysplasia [75]; (Table 1). SBLs, presumably originating from the neural crest, resemble dense, compact bone foci located within the bone marrow cavity, especially in the rachis and sacrum. Since SBLs normally do not produce symptoms, patients do not undergo biopsy, and no molecular studies have been performed on these lesions. Therefore, it is not known whether they follow a “second hit” pattern, like other TSC lesions [75].

The presence of SBLs in TSC patients and localization in the posterior structures of the vertebral bodies require differential diagnosis from osteoblastic metastases. The radiological characteristics of bone lesions detected by CT scan and MRI can help distinguish these bone pathologies [75].

#### Role of *TSC1* and *TSC2* genes in bone phenotype

Studies investigating a possible direct role of *TSC1* and *TSC2* genes in the regulation of bone metabolism are not available. However, the inactivation of the hamartin-tuberin complex causes hyper-activation of mTOR, resulting in uncontrolled cell proliferation and growth that could also occur in bone tissue. Only a small percentage (14%) of patients without *TSC1* or *TSC2* mutations showed SBLs, while mutated patients develop SBLs in 84% of cases (of these, 47.2% have a pathogenic variant of *TSC1* and 52.8% of *TSC2*) [75].

Early post-natal treatment with rapamycin, a mTOR inhibitor, in mice with a neural crest-specific *Tsc1* deletion, prevented the growth of aberrant bone mass, whereas late treatment did not have this effect [76]. These data suggest that the increase of mTOR signal in the neural crest may expand the pool of osteoprogenitor cells at an early stage of the postnatal phase by promoting its proliferation; this is responsible for the increased number of osteoblasts and increase in bone mass in the later postnatal phase. At 1 month, the expanded pool of osteoprogenitor cells is already defined and its maintenance no longer requires an increased activity of mTOR. Therefore, late treatment with rapamycin is not effective in preventing excessive activity of osteogenic cells and consequent acquisition of localized excesses in bone mass. This suggests that it is necessary to identify a treatment window for successful management of bone manifestations in TSC.

### Neurofibromatosis type 1

NF1 is characterized by a pathological triad: 1) multiple benign tumors of the nerves (neurofibromas); 2) pigmented skin lesions (café-au-lait spots and fibrous tumors); and 3) iris hamartomas (Lisch nodules) [67, 68]. Affected individuals have greater susceptibility to the development of benign and malignant tumors, such as neurofibromas, neurofibrosarcomas, gliomas, PHEO, myeloid leukemia, pancreatic and duodenal NETs (especially somatostatinomas) [77, 79]; (Table 1).

NF1 is caused by inactivating mutations of the *NF1* tumor-suppressor gene (17q11.2) (Table 1), encoding neurofibromin 1, a negative regulator of the RAS transduction pathway, which regulates basic functions, such as proliferation, differentiation and apoptosis [77, 78].

The target therapy in NF1 is obviously focused on the RAS pathway, whose inhibition allows to treat multiple manifestations. Most of these drugs are not currently used in clinical practice yet but are subject to clinical trials: one of the most promising is Selumetinib, a MEK inhibitor (a downstream protein in RAS pathway), which has proven effectiveness in neurofibromas and low-grade gliomas. Other drugs that have shown encouraging results are anti angiogenic and mTOR inhibitors [80]. A recent study [81] has highlighted the possibility of restoring the correct RAS pathway via gene delivery, using a panel of adeno-associated virus vectors in peripheral nerve sheath tumor and human Schwann cells. The study showed a dramatically inhibition of the RAS pathway and tumor development, laying the foundations for a possible future gene therapy for NF1 [81].

#### Bone phenotypes in NF1

NF1 manifests various bone lesions (mainly involving long bones and vertebrae), such as kyphoscoliosis, macrocephaly, sphenoid wing dysplasia, and congenital pseudoarthritis of the tibia [82]; (Table 1). These skeletal manifestations affect over 50% of NF1 patients.

Less common skeletal manifestations include local overgrowth, abnormalities of the rib cage, rib fusion, genu varum/valgum, lytic bone lesions, osteosclerosis, absence of the patella, and syndactyly [73]. Bone lesions are mainly congenital and often associated with each other, strongly suggesting that *NF1* has a direct effect on proper skeletal development and correct homeostasis of bone remodeling, and that its inactivating mutations are responsible for the development of NF1 bone complications.

NF1 patients show a higher incidence of osteopenia and osteoporosis compared to the general population [83]. Over 50% of NF1 patients show reduced BMD starting from childhood, not allowing the achievement of correct peak bone mass and predisposing them to a high risk of fragility fractures in adulthood. This reduced BMD can be the cause of long bones bending, mainly in the lower limbs (tibia and fibula).

Pseudarthritis is one of the most severe complications associated with NF1, still without effective therapy. Genes encoding epiregulin (*EREG*) and its receptor (epidermal growth factor receptor 1; *EGFR1*) are overexpressed in cells of the NF1 pseudarthrosis site. *EGFR1* stimulation inhibits osteogenic differentiation. It has been hypothesized that increased *EREG* and *EGFR1* expression in NF1-deficient skeletal progenitors may contribute to their reduced osteogenic differentiation potential. However, studies showed that drugs able to inhibit EGFR1 signaling are unlikely to have a significant benefit for the management of bone non-union in children with NF1 pseudarthrosis [84].

#### Role of *NF1* gene in bone phenotype

Homozygous *Nf1*-KO mice (*Nf1*^*−/−*^) died in utero, while heterozygous mice (*Nf1*^*+/−*^) showed no pathological involvement of bone tissue. Mice with a conditional specific inactivation of *Nf1* in limb skeleton (*Nf1Prx1* mice) showed two main signs: bowing of the tibia and diminished growth, the first due to decreased fitness of the cortical bone, the latter to lower proliferation rates and differentiation defect of chondrocytes [85]. MSC cultures showed increased proliferation and reduced osteogenic differentiation (lower expression of osteocalcin and osteonectin) and mineralization (increased expression of osteopontin). Neurofibromin negatively regulates RAS in osteoblasts [83]; in absence of neurofibromin, the activation of RAS is constitutively maintained, with consequent increased cell growth and reduced cell differentiation.

All these data suggest that neurofibromin is an important modulator of skeletal development and growth, and that somatic loss of the wild type *NF1* allele in osteoprogenitor cells is necessary to develop NF1-associated bone phenotypes.

## Conclusions

Until now, medical and scientific studies on hereditary endocrine syndromes focused on the diagnosis and treatment of tumor manifestations, while associated skeletal and mineral metabolism abnormalities were poorly investigated.

An epidemiological and clinical investigation of bone phenotypes, and their incorporation into the phenotypic description of syndromes, can improve medical knowledge and favor the design of appropriate prevention and management of these complications.

Future studies need to address the genotype-phenotype relationships between gene mutations and severity of bone affections in larger population analyses.

The clinical management of congenital endocrine tumor syndromes can help see the role of “cancer” genes in the regulation of bone development and metabolism in physiological and pathological conditions.

## Data Availability

Not applicable: Data sharing is not applicable to this article as no datasets were generated or analysed during the current study.

## Abbreviations

ALP: Alkaline phosphatase
BMD: Bone mineral density
BM-MSCs: Bone marrow mesenchymal stem cells
CFU-f: Colonies-forming units-fibroblasts
CLA: Cutaneous lichen amyloidosis
CNC: Carney complex
CS: Cowden syndrome
ERα: Estrogen receptor α
FA: Familial acromegaly
FHH: Familial hypocalciuric hypercalcemia
FIPA: Familial isolated pituitary adenoma
FMTC: Familial medullary thyroid carcinoma
FPHPT: Familial primary hyperparathyroidism
FTs: Functioning tumors
GEP-NETs: Neuroendocrine tumors of gastro-entero-pancreatic tract
HD: Hirschsprung disease
HNPGL: Head and neck paraganglioma
HPT-JT: Hyperparathyroidism-Jaw Tumor syndrome
KO mice: Knock-out mice
MEN1: Multiple Endocrine Neoplasia type 1
MEN2 (A and B): Multiple Endocrine Neoplasia type 2 (A and B)
MEN4: Multiple Endocrine Neoplasia type 4
MSCs: Mesenchymal stem cells
MTC: Medullary thyroid carcinoma
NF1: Neurofibromatosis type 1
NFTs: Non-functioning tumors
NLSs: Nuclear localization signals
PGLs: Paragangliomas
PGL/PCC: Hereditary Paraganglioma/Pheochromocytoma syndromes
PHEO: Pheochromocytoma
PHPT: Primary hyperparathyroidism
PTH: Parathyroid hormone
PTH1R: PTH receptor
PTHrP: PTH-related peptide
RCC: Renal cell carcinomas
SBL: Sclerotic bone lesions
TSC: Tuberous Sclerosis Complex
VDR: Vitamin D receptor
VHL: Von Hippel Lindau
